# Palmar Erythema as the Sole Manifestation of COVID-19

**DOI:** 10.7759/cureus.11291

**Published:** 2020-11-02

**Authors:** Balasaraswathy Panambur, Srinivasa B Kakkilaya

**Affiliations:** 1 Dermatology, Spandana Center for Metabolic Medicine, Mangaluru, IND; 2 Internal Medicine, Spandana Center for Metabolic Medicine, Mangaluru, IND

**Keywords:** covid-19, cutaneous manifestations, palmar erythema

## Abstract

The new pandemic COVID-19 is now recognised as a multisystem disease. Variety of skin lesions have been reported in 0.2-20.4% of cases of COVID-19. In some cases of COVID-19, skin lesions have been reported as the initial or the only manifestation. We are reporting a case of bilateral palmar erythema as the sole manifestation of COVID-19 in a 37-year-old female who had a family history of COVID-19 like illness and was later found positive for anti-SARS-CoV-2 antibodies.

## Introduction

COVID-19, caused by SARS-CoV-2 virus, has been spreading across the globe as a pandemic, with more than 37 million cases reported as on second week of October, 2020 [1]. Initially recognised as a respiratory infection, it has since been understood to involve multiple organs. Skin manifestations in COVID-19 patients have been reported even in the initial stages of the pandemic in China, among two of 1099 patients with severe respiratory disease [2], and since then, the reported incidence of skin lesions in COVID-19 cases has been in the range of 0.2-20.4% [3].

Skin manifestations reported in COVID-19 have included erythematous or morbilliform rash, papulosquamous eruption, urticarial lesions, vesicular eruptions, all mainly on the trunk or limbs [4-6], petechiae, retiform purpura [6,7], transient livedo reticularis [8], pernio-like acral lesions [6,9], and enanthems [10].

More recent studies have revealed that skin manifestations can be not only common but be the only manifestation of COVID-19. A study from the UK showed that patients with at least one classical COVID-19 symptom or with swab positivity had significantly higher occurrence of either a body rash (urticarial or vesicular) or an acral rash and that in 17% of swab positive cases, the rash was the initial presentation and in 21%, the rash was the only clinical sign. This report concluded that skin rashes cluster with other COVID-19 symptoms, are predictive of a positive swab test and occur in a significant number of cases, either alone or before other classical symptoms and that recognising rashes was important in identifying new and earlier COVID-19 cases [11].

The pathophysiological relationship of the observed skin lesions to SARS-CoV-2 infection or its severity has not yet been established; the possibilities of the rashes being caused by co-infection with another infection, or by medications used in COVID-19 patients, or by fever or complications such as disseminated intravascular coagulation are also being contemplated [3,12,13].

When SARS-CoV-2 is infecting scores of people during the ongoing pandemic, it is important to look for cutaneous manifestations that may be suggestive of COVID-19 in every patient, whether or not having other classical symptoms of COVID-19 and also to evaluate and report such cases.

## Case presentation

**Figure 1:**
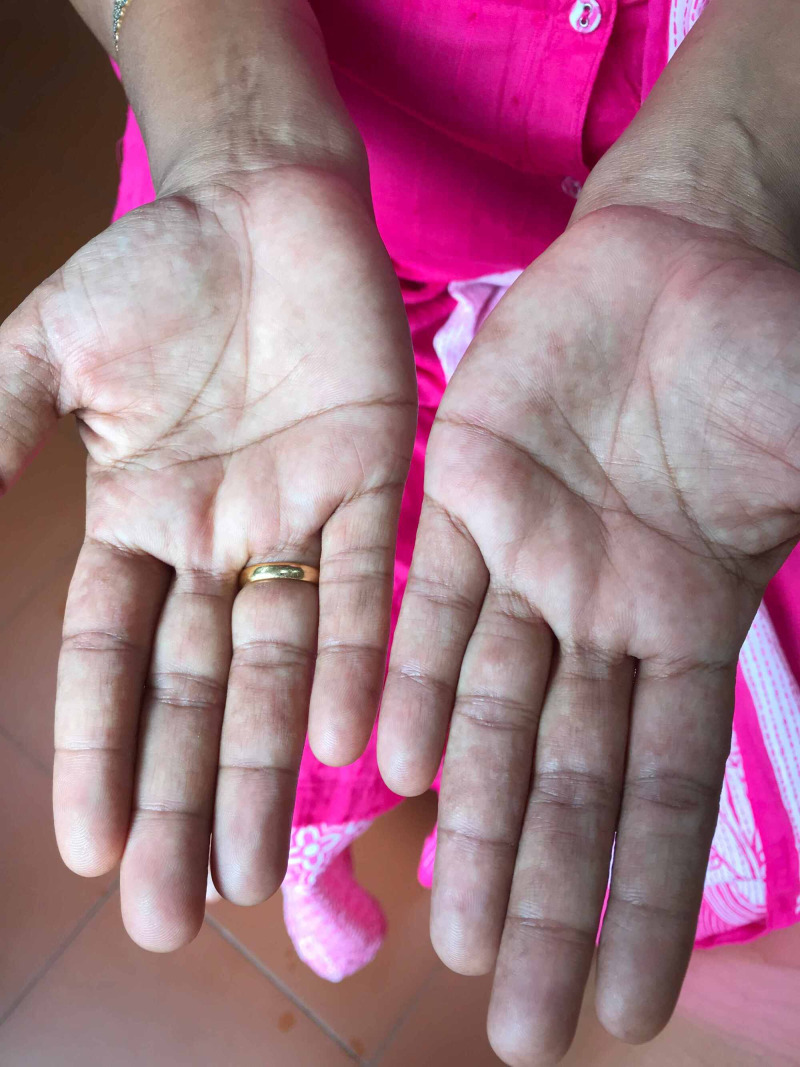
Bilateral palmar erythema

**Figure 2:**
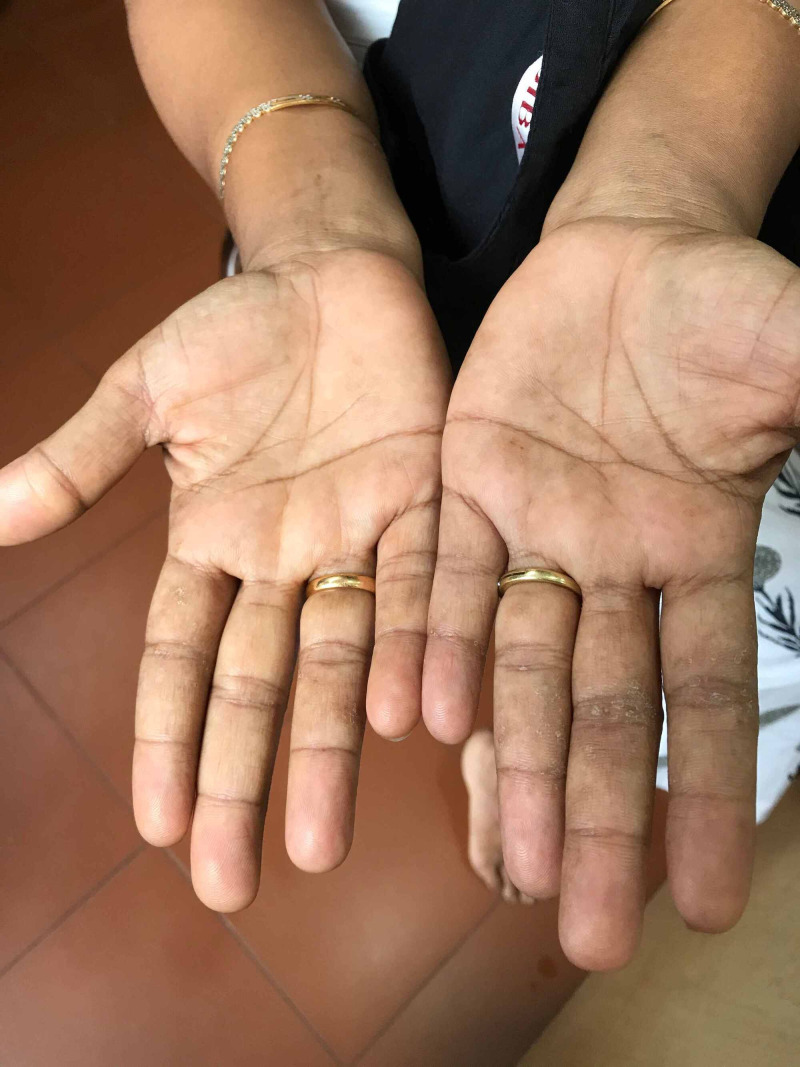
Palmar erythema cleared within 10 days

A 37-year-old lady had earlier presented with pompholyx and disseminated eczema in the month of August, 2020, and was advised patch testing as she had suffered from similar problems several times earlier. When she came for the scheduled skin patch testing on September 15th, 2020, she complained of itching in the palms. On examination, there was erythema of the palms (Figure 1), which was different from the past eruption of pompholyx. There was no skin eruption anywhere else. She had no history of fever, upper respiratory symptoms or any other symptoms suggestive of COVID-19. She did not have any history, symptoms or signs of liver disease, connective tissue disease, thyrotoxicosis, and there was no history of use of any medications. However, there was history of fever, headache and sore throat in her family members, her husband and a daughter, one week prior to her visit, but they hadn’t undergone any test for SARS-CoV-2, and had recovered in 2-3 days. Considering the family history, and with a high degree of suspicion of COVID-19, she was asked to isolate and report after 10 days. On her re-visit on September 23, her palmar erythema had disappeared completely (Figure 2).

She was subjected to COVID-19 (IgM+IgG+IgA) Microlisa test (Double Antigen Sandwich ELISA by J. Mitra & Co, New Delhi, India, with a specificity of 100% and sensitivity of 94-97%) on September 27, 2020 and it was positive with 423 antibody units (normal <9 units). (We preferred this sensitive and specific test for SARS-CoV-2 antibodies as a better indicator of infection than the reverse transcription-polymerase chain reaction (RT-PCR) test). Hence, the self-limiting palmar erythema was considered to be the only manifestation of COVID-19 in her.

## Discussion

Exanthematous and enanthematous lesions are well known in many infections, viral, bacterial, spirochaetal, rickettsial, parasitic and helminthic, and some of these lesions have specific morphologies and are considered as pathognomonic (Koplik’s spots of measles, evanescent rashes of spirochaetal infections, generalised rash of dengue, eschar of rickettsial fever, etc.), leading to diagnosis of the related infections [14,15]. The skin lesions in these infections have been attributed to direct effect of the infectious agent, immune response between infectious agent and antibodies or cell-mediated response to it [15].

The new SARS-CoV-2 pandemic has unfolded in real time since December 2019 and its manifestations have been keenly observed by clinicians all over the world and the reports have been published without any delay. In comparison, the two other corona viruses that were found to cause severe infections, SARS-CoV-1 and MERS, identified in 2003 and 2012 respectively, were not reported to cause any skin manifestations [16,17].

With the volume of information increasing by the day, SARS-CoV-2 that was initially considered as an infection of the respiratory system, soon came to be known as a multisystem illness, including thromboembolic phenomenon. Skin lesions, therefore, are not surprising at all in such an illness, and could, in fact, harbinger or denote different phases of the disease and its underlying pathomechanisms [18].

The skin lesions in COVID-19 have been attributed directly to the virus, or indirectly to vascular changes. Whereas morbilliform rash, petechial rash, erythematous-to-purpuric coalescing macules, widespread urticaria, and varicella-like vesicles may indicate viral exanthems that are immune responses to viral nucleotides, other lesions such as peripheral cyanosis with bullae and dry gangrene, transient unilateral livedo reticularis, and red papules on fingers resembling chilblains may be vasculopathy-related skin manifestations, secondary to systemic consequences caused by COVID-19, especially vasculitis and thrombotic vasculopathy [12].

The skin lesions in COVID-19 may therefore precede the general symptoms or may even be the only sign for a putative infection, serving as early indicators of the disease or of asymptomatic virus carriers. The skin lesions that occur late during infection or even after resolution of main symptoms, might indicate a lack of viral clearance and cascades of immune responses induced by the virus [18]. Therefore, clinicians and especially dermatologists, must keenly look for any possible skin manifestations of COVID-19 in all the patients presenting to a skin clinic.

Our patient was found to have only bilateral palmar erythema, without any other exanthematous or enanthematous lesions, and she did not have any other symptoms suggestive of COVID-19, such as fever, head ache, body ache, sore throat, loss of smell or diarrhoea, nor had she used any medications, systemically or locally. A possibility of COVID-19 as a cause for the palmar erythema was considered out of high index of suspicion, as the infection had been spreading in the town, and on eliciting her family history, it was found that her husband and one of her two daughters had symptoms suggestive of COVID-19 a week earlier. The erythema spontaneously disappeared within 10 days and was clear at her subsequent visit. The positive antibody test confirmed that she did have SARS-CoV-2 infection, and therefore, the palmar erythema can be considered as the sole manifestation of COVID-19 in this patient.

Palmar erythema can be a physiological finding or secondary to systemic pathology. Physiologically, it can occur in at least 30% of pregnant women and it can be associated with diseases such as liver cirrhosis, autoimmune diseases (such as rheumatoid arthritis and lupus erythematosus), thyrotoxicosis, diabetes mellitus, infections (brucellosis, trichinellosis, bacterial endocarditis), Kawasaki disease, certain neoplasms or drugs [19]. Palms are known to have higher density of arteriovenous shunts and palmar erythema has been attributed to alterations in the function of the skin and its microvasculature, such as increased dilatation of capillaries and superficial arterial and venous plexi in the palm, which in turn have been linked to estrogen, bradykinin and other vasoactive substances or angiogenic factors [19]. Our patient did not have any history or manifestations of other possible causes for palmar erythema, and the palmar erythema in this case was self-limiting, disappearing within 10 days of being detected. Therefore, the palmar erythema in our patient, seropositive for COVID-19, is likely to be due to the vascular pathological changes caused by COVID-19.

## Conclusions

Palmar erythema may be the only clinical manifestation of COVID-19. During the community transmission of novel infections such as SARS-CoV-2 that can cause cutaneous lesions, dermatologists must examine all their patients thoroughly for skin lesions that may be suggestive of such infections and thereby help in identifying such infections and in treating and controlling them.
